# Supplementary material for: Accelerating target deconvolution for therapeutic antibody candidates using highly parallelized genome editing
Source: Nat Commun. 2021 Feb 24;12:1277. doi: 10.1038/s41467-021-21518-4 (PMC7904777; doi:10.1038/s41467-021-21518-4)
Supplement: Supplementary file 1 — Supplementary Information [file 41467_2021_21518_MOESM1_ESM.pdf]

Accelerating target deconvolution for therapeutic antibody candidates using highly parallelized genome editing

Supplementary Figure 1

Flow cytometry data showing antibody binding to test cells. Treg mAbs (red tones), prostate cancer mAbs (blue tones), TAM mAbs (yellow tones), control mAbs (turquoise tones) and isotype controls (grey). **(a)** Binding to Jurkat cells for unconjugated mAbs detected with anti-human IgG-APC. **(b)** Binding to H9 cells for unconjugated mAbs detected with anti-human IgG-APC. **(c)** Binding to activated CD4 cells for unconjugated mAbs detected with anti-human IgG-APC. **(d)** Binding to THP1 cells for biotinylated mAbs detected with streptavidin-APC. **(e)** Binding to PMA-polarized THP1 cells for biotinylated antibodies detected with streptavidin-APC. **(f,g)** Binding to Jurkat cells for control antibodies conjugated with PE-Cy7 and APC, respectively.

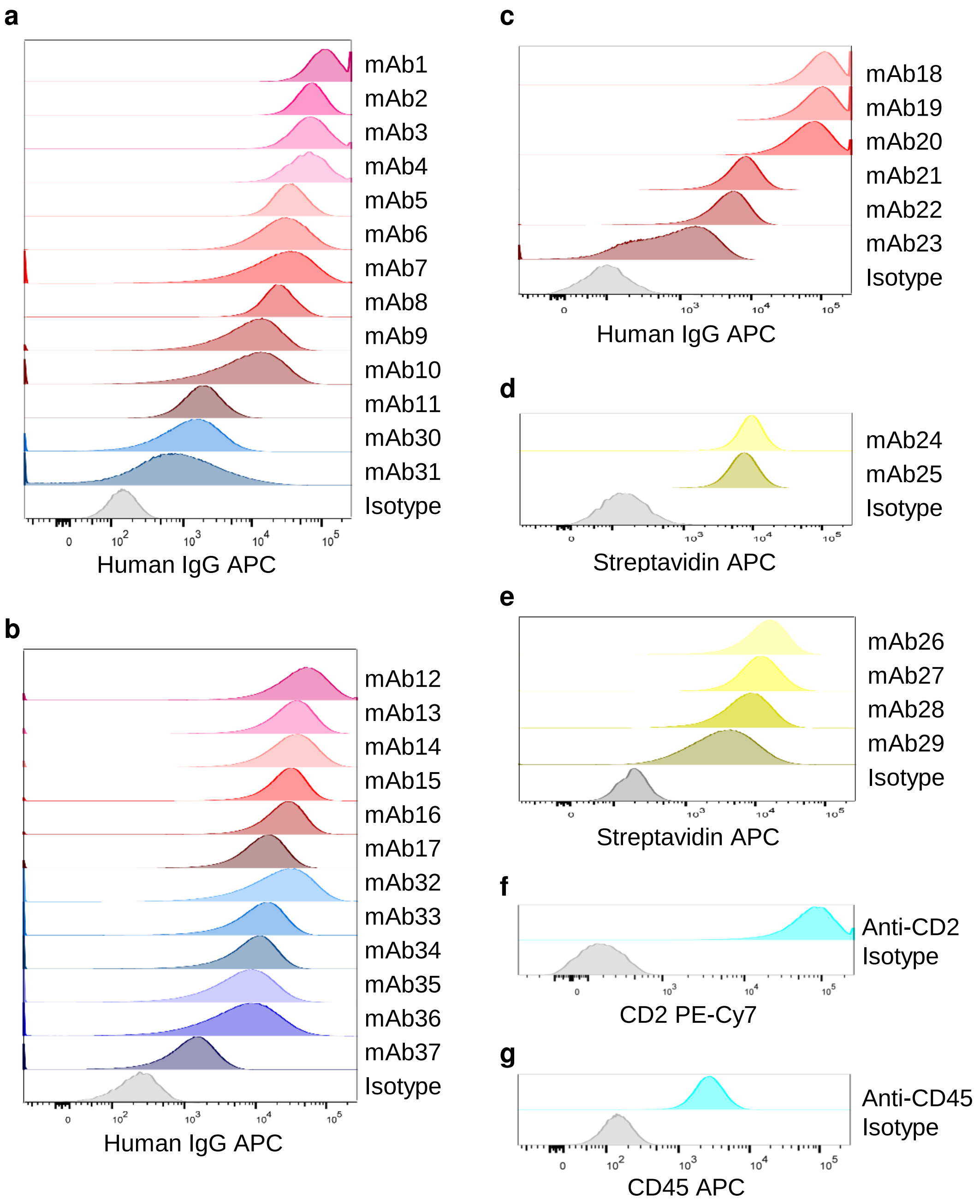

Supplementary Figure 2

MAGeCK scores (y-axis) and FDR (x-axis). Red dots indicate prioritized target genes, pink MHC class I dependency genes. Blue circles in mAb26, mAb33 and mAb34 indicate integrin chaperone genes *HSP90B1* and *MESDC2*, and regulator gene *MAP2K1*. Blue circle in mAb37 indicates *IRF2*, influencing IL15RA expression. Source data are provided as a Source Data file.

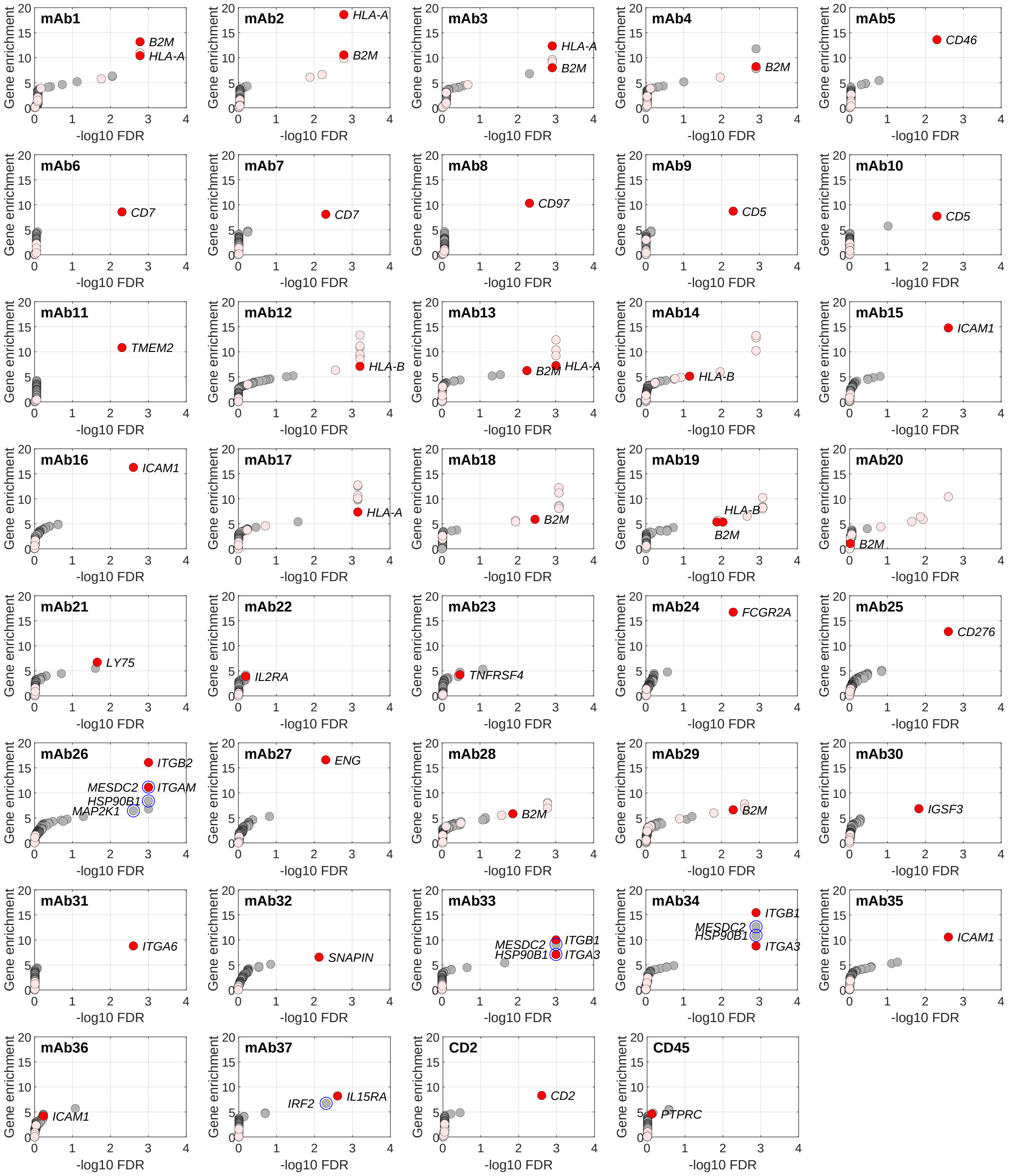

Supplementary Figure 3

Target validation. **(a)** ELISA data with mAb and target (red) with mAb and non-target (black) and IgG1 isotype control and target (blue) indicated. **(b)** Blocking data with target cells incubated with mAb (red), IgG1 isotype control (blue) or mAb after blocking with polyclonal antibody to the identified target protein (dotted). **(c)** Transfection data for CHO (mAb8) or HEK (mAb11 and mAb26) cells transfected with cDNA encoding target protein, incubated with mAb (red) or IgG isotype control (blue). Also shown is mock-transfected cells incubated with mAb (dotted). As shown, we validated the specificities of 23 of the 24 mAbs identified as non-MHC antibodies, and 9 of the 13 mAbs identified as MHC class I antibodies (**Fig. 2a** and **Supplementary Fig. 2**). Source data are provided as a Source Data file.

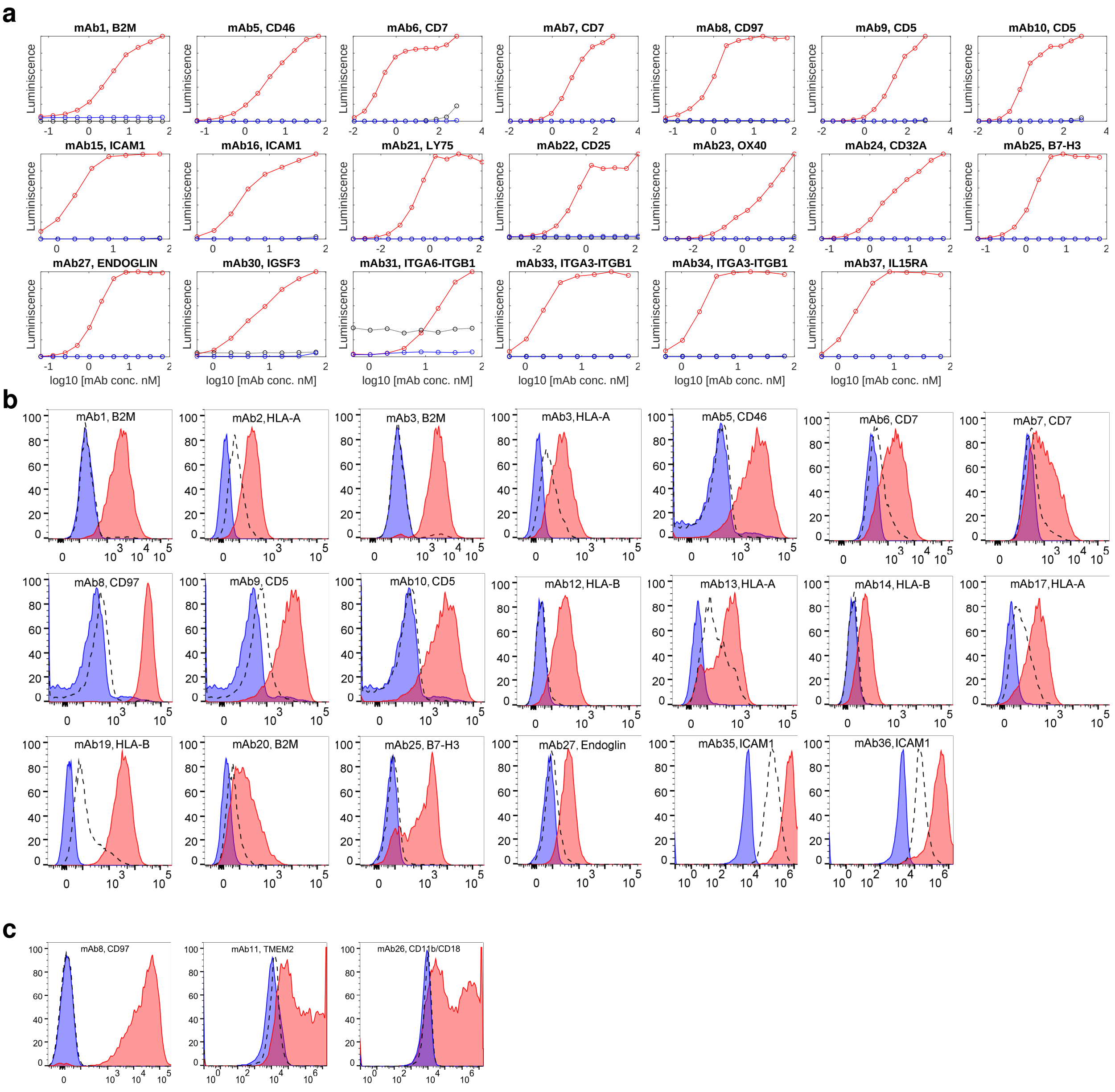

Supplementary Figure 4

Sequential gating path for cell sorting. First, we gated cells based on size (forward scatter area, FSC-A, and side scatter area, SSC-A). Second, we gated live (SYTOX Green negative) cells. Third, we gated antigen-negative cells as the 1.5 to 2% most APC-negative cells and control cells as the 20% most APC-positive cells.

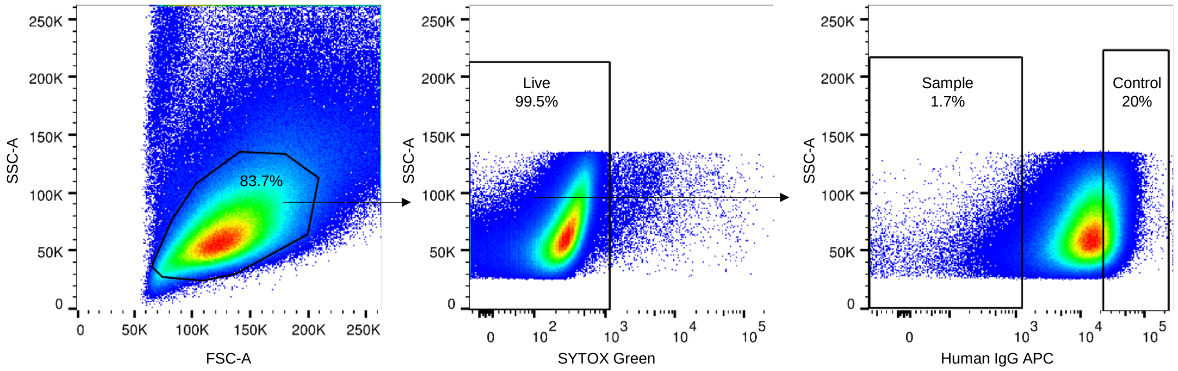

Supplementary Figure 5

Example of inter-individual variation in antigen expression between donors of primary *in vitro*-activated CD4^{+} T cells. The broad staining pattern in Donor D also signals the presence of intra-individual phenotypic variation. This example illustrates binding of antibody mAb23. Unconjugated antibody detected with anti-human IgG-APC.

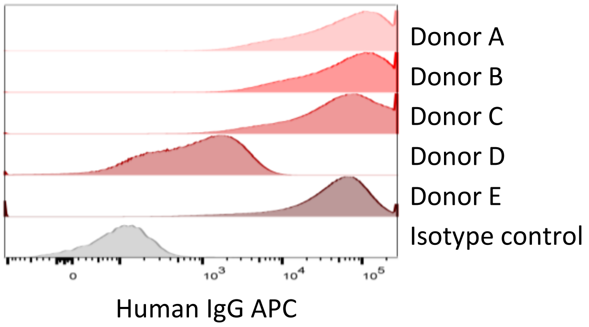

Supplementary Table 1

Summary of target gene selection.

mAb	Test cell	Library	Control cells	Pre-enriched	Selection criterion I[1]	II[2]	III[3]	Prioritized genes
mAb1	Jurkat	GeCKO	Unsorted	x	x	x		*B2M*, *HLA-A*
mAb2	Jurkat	GeCKO	Unsorted	x	x	x		*HLA-A*, *B2M*
mAb3	Jurkat	GeCKO	Unsorted	x	x	x		*HLA-A*, *B2M*
mAb4	Jurkat	GeCKO	Unsorted	x	x	x		*B2M*
mAb5	Jurkat	GeCKO	Unsorted	x	x			*CD46*
mAb6	Jurkat	GeCKO	Unsorted	x	x			*CD7*
mAb7	Jurkat	GeCKO	Unsorted	x	x			*CD7*
mAb8	Jurkat	GeCKO	Unsorted	x	x			*CD97*
mAb9	Jurkat	GeCKO	Unsorted	x	x			*CD5*
mAb10	Jurkat	GeCKO	Unsorted	x	x			*CD5*
mAb11	Jurkat	GeCKO	Unsorted	x	x			*TMEM2*
mAb12	H9	Brunello	Unsorted	x	x	x		*HLA-B*
mAb13	H9	Brunello	Positive	x	x	x		*HLA-A*, *B2M*
mAb14	H9	Brunello	Positive	x		x		MHC class I
mAb15	H9	Brunello	Unsorted	x	x			*ICAM1*
mAb16	H9	Brunello	Unsorted	x	x			*ICAM1*
mAb17	H9	Brunello	Unsorted	x	x	x		*HLA-A*
mAb18	$CD4^+$	Brunello	Positive		x	x		*B2M*
mAb19	$CD4^+$	Brunello	Positive		x	x		*B2M*, *HLA-B*
mAb20	$CD4^+$	Brunello	Positive			x		MHC class I
mAb21	$CD4^+$	Brunello	Positive		x			*LY75*, *LY75-CD302*
mAb22	$CD4^+$	Brunello	Positive				x	*IL2RA*
mAb23	$CD4^+$	Brunello	Positive				x	*TNFRSF4*
mAb24	THP-1	Brunello	Positive	x	x			*FCGR2A*
mAb25	THP-1	Brunello	Positive	x	x			*CD276*
mAb26	THP-1	Brunello	Positive		x			*ITGB2*, *ITGAM*
mAb27	THP-1	Brunello	Positive		x			*ENG*
mAb28	THP-1	Brunello	Positive		x	x		*B2M*
mAb29	THP-1	Brunello	Positive		x	x		*B2M*
mAb30	Jurkat	GeCKO	Positive	x	x			*IGSF3*
mAb31	Jurkat	GeCKO	Positive	x	x			*ITGA6*
mAb32	H9	Brunello	Unsorted	x	x			*SNAPIN*
mAb33	H9	Brunello	Unsorted	x	x			*ITGB1*, *ITGA3*
mAb34	H9	Brunello	Unsorted	x	x			*ITGB1*, *ITGA3*
mAb35	H9	Brunello	Unsorted	x	x			*ICAM1*
mAb36	H9	Brunello	Positive	x			x	*ICAM1*
mAb37	H9	Brunello	Unsorted	x	x			*IL15RA*
CD2	Jurkat	GeCKO	Unsorted	x	x			*CD2*
CD45	Jurkat	GeCKO	Unsorted	x			x	*PTPRC*

[1]Membrane protein genes with significant enrichment score in antigen-negative cells (MAGeCK false discovery rate < 5%). [2]Enrichment of MHC class I dependencies (Bonferroni-adjusted one-sided Wilcoxon $P < 0.05$ for MHC class I dependency genes versus other genes). [3]Most enriched membrane protein genes, as fall-back criterion when no gene satisfied criterion I or II.

Supplementary Table 2

Summary of methods used to validate antibody specificities in **Supplementary Fig. 3.**

Antibody	Target	Validation method			
		ELISA[1]	Block[2]	Overexpression[3]	MHC-I dependencies[4]
mAb1	MHC class I	x, B2M	x, B2M		
mAb2	MHC class I		x, HLA-A		
mAb3	MHC class I		x, HLA-A, B2M		
mAb4	MHC class I				x
mAb5	CD46	x	x		
mAb6	CD7	x	x		
mAb7	CD7	x	x		
mAb8	CD97	x	x	x	
mAb9	CD5	x	x		
mAb10	CD5	x	x		
mAb11	TMEM2			x	
mAb12	MHC class I		x, HLA-B		
mAb13	MHC class I		x, HLA-A		
mAb14	MHC class I		x, HLA-B		
mAb15	ICAM1	x			
mAb16	ICAM1	x			
mAb17	MHC class I		x, HLA-A		
mAb18	MHC class I				x
mAb19	MHC class I		x, HLA-B		
mAb20	MHC class I		x, B2M		
mAb21	LY75	x			
mAb22	CD25	x			
mAb23	OX40	x			
mAb24	CD32A	x			
mAb25	B7-H3	x	x		
mAb26	MAC1			x	
mAb27	ENDOGLIN	x	x		
mAb28	MHC class I				x
mAb29	MHC class I				x
mAb30	IGSF3	x			
mAb31	ITGA6	x			
mAb32	SNAPIN				
mAb33	VLA3	x			
mAb34	VLA3	x			
mAb35	ICAM1		x		
mAb36	ICAM1		x		
mAb37	IL15RA	x			

[1]Enzyme-linked immunsorbent assay with recombinant target protein. [2]Inhibition of mAb binding to target cells by blocking with competing polyclonal antibody against the identified target. [3]Overexpression of cDNA encoding the identified target in cell lines, followed by flow cytometry. [4]Significant enrichment of MHC class I dependency genes in CRISPR data (one-sided Wilcoxon test Bonferroni-adjusted $P<0.05$).

Supplementary Table 3

sgRNA amplification primers.

sgRNA primer	Sequence
Forward	5′-AATGATACGGCGACCACCGAGATCTACACTCTTTCCCTACACGACGCTCTTCCGATCT - (1–12 bp variable length sequence) - tcttgtggaaaggacgaaacaccg -3′
Reverse	5′-CAAGCAGAAGACGGCATACGAGAT – 8nt Index – GTGACTGGAGTTCAGACGTGTGCTCTTCCGATCTtgtgggcgatgtgcgctctg -3′

Upper case - sequencing adaptors, lower case - CRISPRv2 primers

Supplementary Table 4

Reagents used for validation of antibody specificity.

Antigen	Recombinant protein	Polyclonal antibody
HLA-A		MyBioSource #MBS8245132
HLA-B		MyBioSource #MBS2522514<br>Nordic BioSite #LS-C308249
B2M	Sino Biological #11976-H08H	Sino Biological #11976-RP02
CD5	Sino Biological #11027-H08H	R&D Systems #AF1636
CD7	Sino Biological #11028-H08H	R&D Systems #AF7579
CD46	Sino Biological #12239-H08H	R&D Systems #AF2005
CD97	Sino Biological #11280-H02H	R&D Systems #AF2529
ICAM1	R&D Systems #720-IC	Sino Biological #10346-T26
LY75	Sino Biological #16490-H08H	
CD25	R&D Systems #1020-RL-050	
ox40	In-house produced	
CD32A	Sino Biological #10374-H08H	
B7-H3	Sino Biological #11188-H08H	Sino Biological #11188-T24
ENDOGLIN	Sino Biological #10149-H08H	Sino Biological #10149-T26
IGSF3	Sino Biological #11290-H08H	Sino Biological #11290-T24
ITGA6	Sino Biological #CT013-H2508H	
ITGA3/ITGB1	R&D Systems #2840-A3	
IL15RA	R&D Systems #7194-IR	
