# Supplementary material for: Accelerating target deconvolution for therapeutic antibody candidates using highly parallelized genome editing
Source: Nat Commun. 2021 Feb 24;12:1277. doi: 10.1038/s41467-021-21518-4 (PMC7904777; doi:10.1038/s41467-021-21518-4)
Supplement: Supplementary file 2 — Reporting Summary [file 41467_2021_21518_MOESM2_ESM.pdf]

nature research

Corresponding author(s): Björn Nilsson
Last updated by author(s): Jan 25, 2021

Reporting Summary

Nature Research wishes to improve the reproducibility of the work that we publish. This form provides structure for consistency and transparency in reporting. For further information on Nature Research policies, see our Editorial Policies and the Editorial Policy Checklist.

Statistics

For all statistical analyses, confirm that the following items are present in the figure legend, table legend, main text, or Methods section.

n/a	Confirmed	
☐	☒	The exact sample size (n) for each experimental group/condition, given as a discrete number and unit of measurement
☐	☒	A statement on whether measurements were taken from distinct samples or whether the same sample was measured repeatedly
☐	☒	The statistical test(s) used AND whether they are one- or two-sided<br>*Only common tests should be described solely by name; describe more complex techniques in the Methods section.*
☒	☐	A description of all covariates tested
☐	☒	A description of any assumptions or corrections, such as tests of normality and adjustment for multiple comparisons
☒	☐	A full description of the statistical parameters including central tendency (e.g. means) or other basic estimates (e.g. regression coefficient) AND variation (e.g. standard deviation) or associated estimates of uncertainty (e.g. confidence intervals)
☐	☒	For null hypothesis testing, the test statistic (e.g. F, t, r) with confidence intervals, effect sizes, degrees of freedom and P value noted<br>*Give P values as exact values whenever suitable.*
☒	☐	For Bayesian analysis, information on the choice of priors and Markov chain Monte Carlo settings
☒	☐	For hierarchical and complex designs, identification of the appropriate level for tests and full reporting of outcomes
☒	☐	Estimates of effect sizes (e.g. Cohen's d, Pearson's r), indicating how they were calculated

Our web collection on statistics for biologists contains articles on many of the points above.

Software and code

Policy information about availability of computer code

Data collection	BD FACSDiva Software v.8.0.2 for flow cytometry and FACS<br>Illumina NextSeq 500 for acquiring read sequences<br>bcl2fastq v.2.20.0.422, for basecalling and demultiplexing<br>Tecan Magellan 3.0 for ELISA
Data analysis	FlowJo v.10.4.2 for analysis of flow cytometry data<br>Agilent 2100 Expert v.B.02.08.SI648 (SR2) for quality control of PCR products<br>FastQC v.0.11.8. for quality control of sequence data<br>MAGeCK v.0.5.9.2 for analysis of sequence data from CRISPR screens<br>RenderCat v1.0 for gene essentiality testing

For manuscripts utilizing custom algorithms or software that are central to the research but not yet described in published literature, software must be made available to editors and reviewers. We strongly encourage code deposition in a community repository (e.g. GitHub). See the Nature Research guidelines for submitting code & software for further information.

Data

Policy information about availability of data

All manuscripts must include a data availability statement. This statement should provide the following information, where applicable:

- Accession codes, unique identifiers, or web links for publicly available datasets
- A list of figures that have associated raw data
- A description of any restrictions on data availability

The data that support the findings of this study are available from the corresponding author upon reasonable request.

Field-specific reporting

Please select the one below that is the best fit for your research. If you are not sure, read the appropriate sections before making your selection.

☒ Life sciences ☐ Behavioural & social sciences ☐ Ecological, evolutionary & environmental sciences

For a reference copy of the document with all sections, see nature.com/documents/nr-reporting-summary-flat.pdf

Life sciences study design

All studies must disclose on these points even when the disclosure is negative.

Sample size	We applied our approach to 39 test antibodies, including 37 with unknown specificity and 2 with known specificity. These antibodies were selected because they were readily available and expected to target a broad range of different antigens. While we did not perform a formal power calculation, the sample size we ended up was sufficient to demonstrate a high success rate.
Data exclusions	No data were excluded.
Replication	We applied our approach to 3 to 5 replicates per test antibody. All attempts were successful, and we saw similar results between replicates.
Randomization	Mammalian cells used in this work were grown under identical conditions; no randomization was used
Blinding	The CRISPR screens were conducted blindly by definition since the antibody targets were not known beforehand.

Reporting for specific materials, systems and methods

We require information from authors about some types of materials, experimental systems and methods used in many studies. Here, indicate whether each material, system or method listed is relevant to your study. If you are not sure if a list item applies to your research, read the appropriate section before selecting a response.

Materials & experimental systems

n/a	Involved in the study	
☐	☒	Antibodies
☐	☒	Eukaryotic cell lines
☒	☐	Palaeontology and archaeology
☒	☐	Animals and other organisms
☐	☒	Human research participants
☒	☐	Clinical data
☒	☐	Dual use research of concern

Methods

n/a	Involved in the study	
☒	☐	ChIP-seq
☐	☒	Flow cytometry
☒	☐	MRI-based neuroimaging

Antibodies

Antibodies used

Monoclonal antibodies for flow cytometry: anti-CD2 PE-Cy7 (BD Biosciences, Clone S5.2, #335821), anti-CD45 APC (BD Biosciences, Clone HI30, #555485).

Secondary antibodies: anti-h-IgG APC (Jackson ImmunoResearch #109-136-098), Anti-human Fab fragment-HRP (Jackson Immunoresearch #109-036-006).

Polyclonal antibodies for blocking experiments: anti-HLA-A (MyBioSource #MBS8245132), anti-HLA-B (MyBioSource #MBS2522514), anti-HLA-B (Nordic BioSite #LS-C308249), anti-B2M (Sino Biological #11976-RP02), anti-CD5 (R&D Systems #AF1636), anti-CD7 (R&D Systems #AF7579), anti-CD46 (R&D Systems #AF2005), anti-CD97 (R&D Systems #AF2529), anti-ICAM1 (Sino Biological #10346-T26), anti-B7-H3 (Sino Biological #11188-T24), anti-Endoglin (Sino Biological #10149-T26), anti-IGSF3 (Sino Biological #11290-T24).

	37 test mAbs, mAb 1-37, human IgG1 antibodies isolated from the n-CoDeR phage display library in three on-going projects (Semmerich et al., Jaensson-Gyllenbäck et al. and Mattsson et al.; more details in manuscript).
Validation	CD2 and CD45 antibodies used for flow cytometry were validated by suppliers for reactivity species (human) and application (flow cytometry).<br><br>Polyclonal antibodies used for flow cytometry blocking experiments were validated by suppliers for reactivity species (human). Antibodies from R&D Systems targeting CD5, CD7, CD46 and CD97 were also validated by suppliers for application (flow cytometry).<br><br>The 37 test mAbs were validated for species (human) and application (flow cytometry) by the authors (supplementary Fig. 1).

Eukaryotic cell lines

Policy information about cell lines

Cell line source(s)	293T/17 (ATCC #CRL-11268), CHO-S (Thermo Fisher Scientific #R80007), Jurkat (DSMZ #ACC 282), H9 (ATCC #HTB-176) and THP-1 (ECACC #88081201).
Authentication	None of the cell lines were autheticated.The cell lines were only used for production of virus or antibodies, or for being antigen positive. Hence authentication was not relevant.
Mycoplasma contamination	The cell lines were not tested for mycoplasma contamination.
Commonly misidentified lines (See ICLAC register)	No misidentified cell lines were used in this study.

Human research participants

Policy information about studies involving human research participants

Population characteristics	Random blood donors, which means the subjects can be aged 18 to ~70 years and have no known serious diseases that affect their eligibility to give blood.
Recruitment	Random blood donors. No biases expected.
Ethics oversight	Ethics Committee of Skåne University Hospital (2010/356).

Note that full information on the approval of the study protocol must also be provided in the manuscript.

Flow Cytometry

Plots

Confirm that:

☒ The axis labels state the marker and fluorochrome used (e.g. CD4-FITC).

☒ The axis scales are clearly visible. Include numbers along axes only for bottom left plot of group (a 'group' is an analysis of identical markers).

☒ All plots are contour plots with outliers or pseudocolor plots.

☒ A numerical value for number of cells or percentage (with statistics) is provided.

Methodology

Sample preparation	CD4+ T cells were isolated from buffy coats from healthy donors using gradient density centrifugation (Ficoll Paque PLUS, GE Healthcare) and CD4 Microbeads (Miltenyi Biotec). Purified cells were activated using Dynabeads Human T-Activator CD3/CD28. Cell lines were transduced with lentiviral CRISPR/Cas9 libraries
Instrument	BD LSRFortessa (BD Biosciences), BD FACSAria Fusion (BD Biosciences)
Software	BD FACSDiva Software v.8.0.2, FlowJo Software v.10.4.2
Cell population abundance	After one sort, the fraction of fluorescence-negative cells were typically 2-5%. After two sorts, the fraction had increased to >20% in most cases. The purity was determined by flow cytometry
Gating strategy	Cells were gated on FCS/SSC followed by gating live cells (SYTOX green negative). The 1.5-2% least fluorescent (antigen-negative) cells were sorted for each test antibody, and the 20% most fluorescent cells were sorted as control.

☒ Tick this box to confirm that a figure exemplifying the gating strategy is provided in the Supplementary Information.
